# Supplementary figures and images for: Contribution of nosocomial transmission to Klebsiella pneumoniae neonatal sepsis in Africa and South Asia: An observational study of infection clusters inferred from pathogen genomics and temporal data
Source: PLoS Med. 2026 May 13;23(5):e1005077. doi: 10.1371/journal.pmed.1005077 (PMC13186336; doi:10.1371/journal.pmed.1005077)

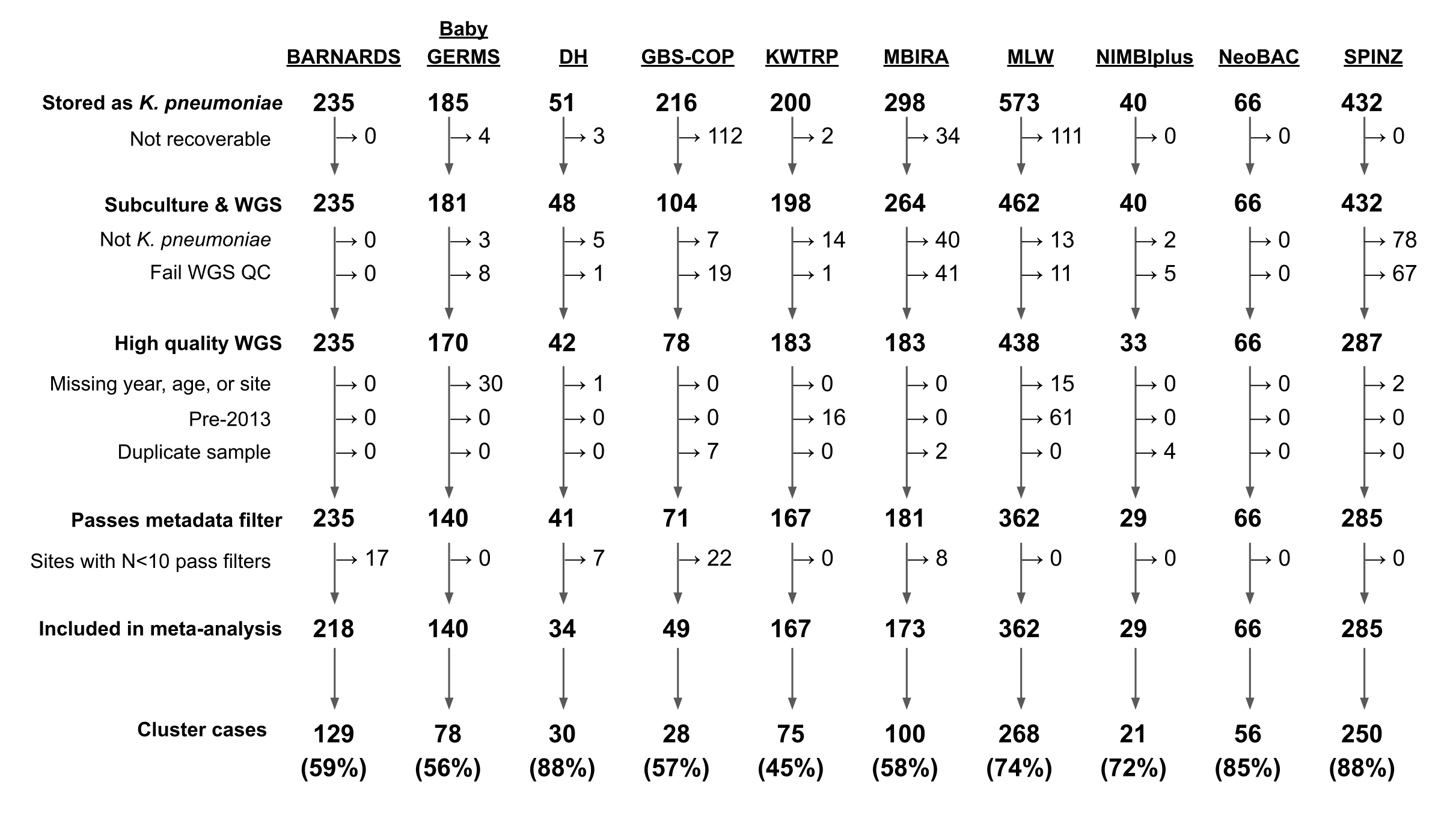

Supplement: S1 Fig — All numbers shown represent the number of isolates. For the line ‘Sites with N<10 pass filters’, the numbers represent the total number of isolates excluded for all excluded sites. (TIF) [file pmed.1005077.s011.tif]

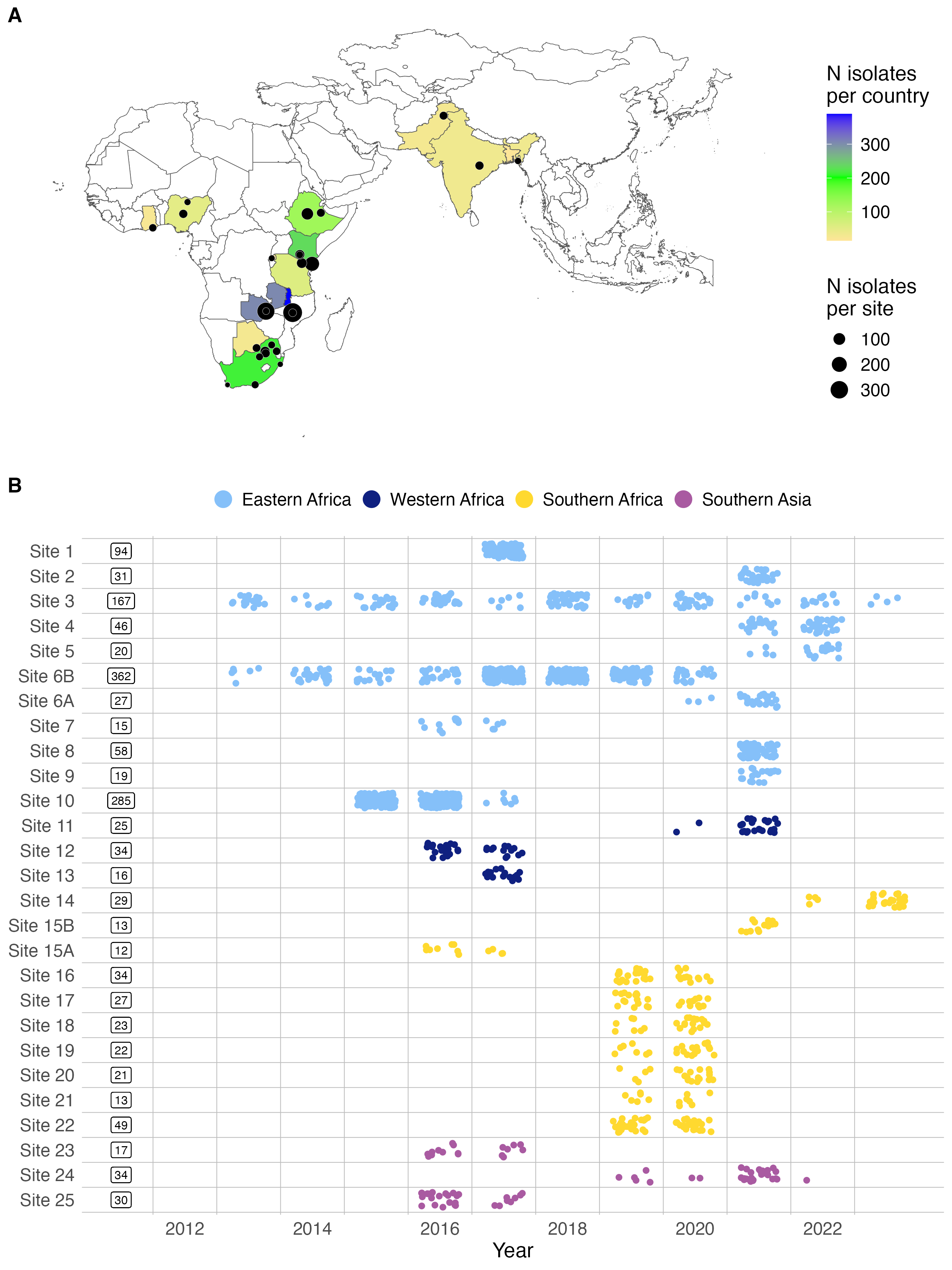

Supplement: S2 Fig — (A) Location of study sites. Each point represents a unique site and point sizes indicate the number of isolates included per site, as per figure legend. Country colours indicate the number of isolates included per country, as per figure legend. The map was generated using the rnaturalearth (version 1.0.1) package in R. Base map source: Natural Earth, https://www.naturalearthdata.com/downloads/10m-cultural-vectors/, accessed using rnaturalearth R package v1.0.1, terms of use: https://www.naturalearthdata.com/about/terms-of-use/. (B) Plot shows sampling dates for all isolates included per site. Each point represents a unique isolate included per site and numbers inside the box represent the number of isolates per site. Points are jittered along the y-axis to aid visibility. (TIF) [file pmed.1005077.s012.tif]

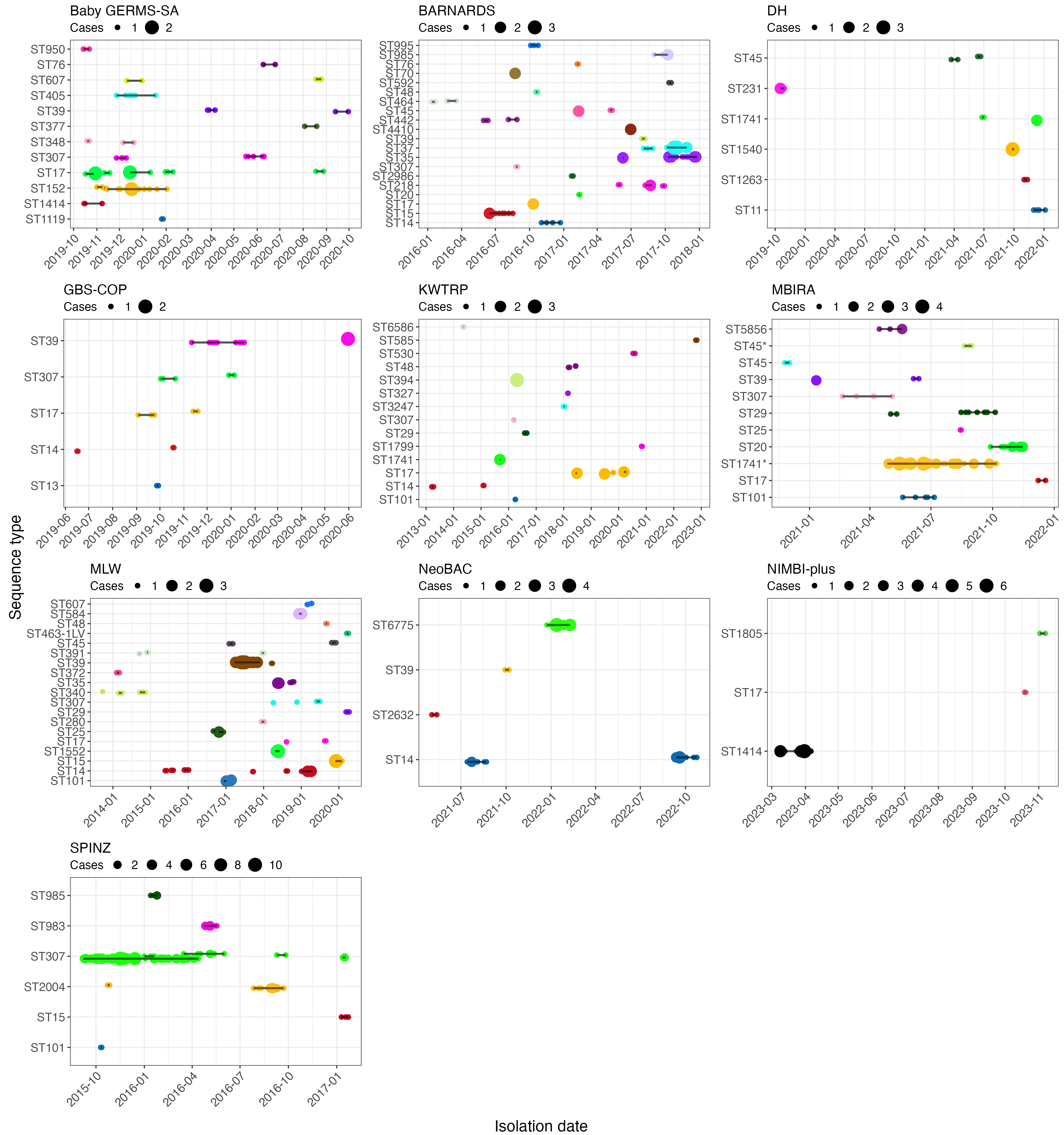

Supplement: S3 Fig — Each point represents one or more cases isolated on specific dates. Points are coloured according to sequence type. Clusters are represented as groups of cases (points) linked by horizontal lines. Clusters belonging to the same sequence type are jittered along the y-axis to allow visibility of overlapping clusters. ST – sequence type. * – includes single locus variants of the respective STs. (TIF) [file pmed.1005077.s013.tif]

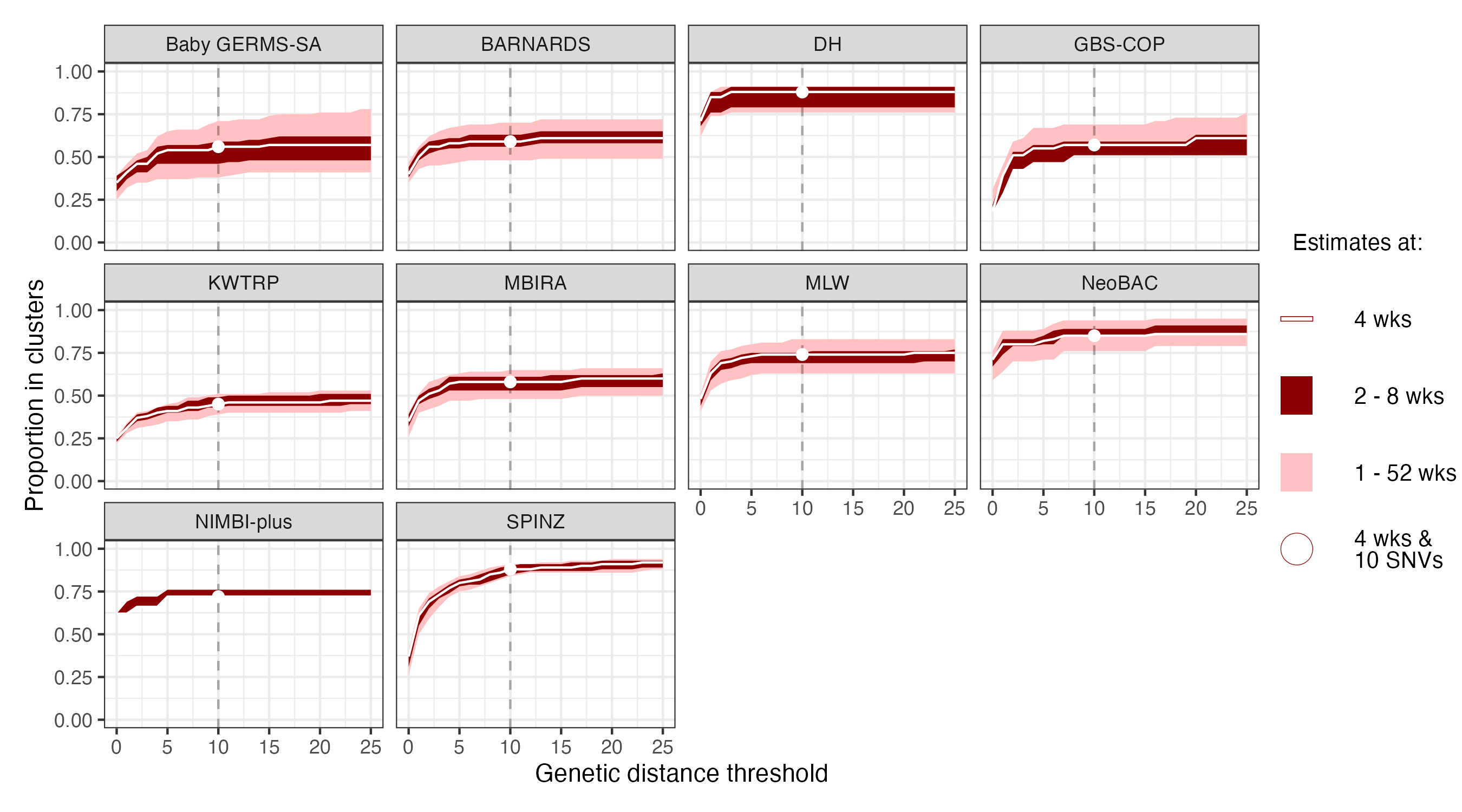

Supplement: S4 Fig — The sub-panels show estimates for individual study datasets at different combinations of genetic distance threshold (x-axis) and temporal distance threshold ranges (as per figure legend). (TIF) [file pmed.1005077.s014.tif]
